# Generalized cystic lymphangiomatosis incidentally diagnosed in an asymptomatic adult: Imaging findings of a very rare case

**DOI:** 10.1016/j.radcr.2023.10.017

**Published:** 2023-10-28

**Authors:** Zhale Tabrizi, Adeleh Dadkhah, Shakiba soleimani, Maryam Moaddab

**Affiliations:** a Department of Radiology, Iran University of Medical Science, Tehran, Iran; b Department of Radiology, Hasheminejad Kidney Center (HKC), Iran University of Medical Science, Tehran, Iran

**Keywords:** Lymphangioma, Lymphangiomatosis, Generalized lymphangiomatosis, Computed tomography scan, Magnetic resonance imaging cytology

## Abstract

Lymphangiomas are benign lesions of vascular origin with lymphatic differentiation, most commonly found in the head and neck. Generalized lymphangiomatosis is a very rare condition in adults, which is characterized by a diffuse proliferation of lymphatic vessels. The lymphangioma is composed of lymphatic endothelium-lined cystic spaces. This condition can be histologically differentiated from other vascular disorders such as cavernous or capillary hemangioma. However, many cases of lymphangioma can be confused with other vascular disorders, because of overlapping histologic findings. radiologic examinations, such as CT scan and MR imaging, are useful for assessing the morphologic feature and also the extent of disease, it is important to know the radiologic findings of generalized lymphangiomatosis. In this paper, we report a case of generalized lymphangiomatosis in a 42-year-old male who presented with left flank pain and hematuria. The first differential diagnosis was renal colic; hence he underwent an abdominopelvic computed tomography scan (CT scan). In the performed CT scan multiple cystic lesions were seen in the liver and spleen. Also, lytic lesions were seen in bones. CT-guided biopsy was performed and the result was compatible with generalized lymphangiomatosis, confirmed by cytology. Generalized lymphangiomatosis is a rarely reported disease in children and young adults. Delayed diagnosis in older patients or misdiagnosis is common due to its rarity and nonspecific clinical presentation. Different imaging modalities can incidentally diagnose the disease in asymptomatic patients. So radiologists should be aware of the disease manifestations in imaging modalities to diagnose the disease sooner and help the clinician start the therapy if needed.

## Background

Lymphangiomatosis is a rare benign proliferation and dilation of the lymphatic channels most commonly found in the head and neck. They can originate from any tissue except ocular and neural tissue that can involve multiple organs with a variety of clinical presentations. This condition may involve soft tissues, bone structures, and various organs in the body such as the liver, spleen, mediastinum, and lungs [1], [2], [3]. Despite children, generalized cystic lymphangiomatosis is extremely rare in adults [4]. The underlying pathogenetic mechanism is unclear, but it is generally considered a congenital malformation of the lymphatic system coexisting with alterations in the circulatory dynamics of the lymph [5]. These dilated lymphatic channels are mostly chyle-filled [6]. This condition can be histologically differentiated from other vascular disorders such as cavernous or capillary hemangioma. However, many cases of lymphangioma can be confused with other vascular disorders, because of the overlap between these condition's histologic findings. Radiologic examinations, such as CT scan and MR imaging, are useful for assessing the morphologic feature and also the extension of the disease, it is important to know the radiologic findings of generalized lymphangiomatosis [7]. However, clinical examination is also helpful. The clinical presentation depends on whether the lesions are symptomatic or not. The lesions can be detected incidentally in an asymptomatic patient, or in a patient with a complication (for example pathologic fractures in symptomatic patients.) [8].

This condition occurs with an equal gender prevalence [2]. We report a very rare case of incidentally diagnosed generalized lymphangiomatosis involving the liver, spleen, mediastinum, vertebrae, and iliac bones in a 42-year-old male, which was subsequently confirmed by histological findings.

## Case presentation

**Fig. 1:**
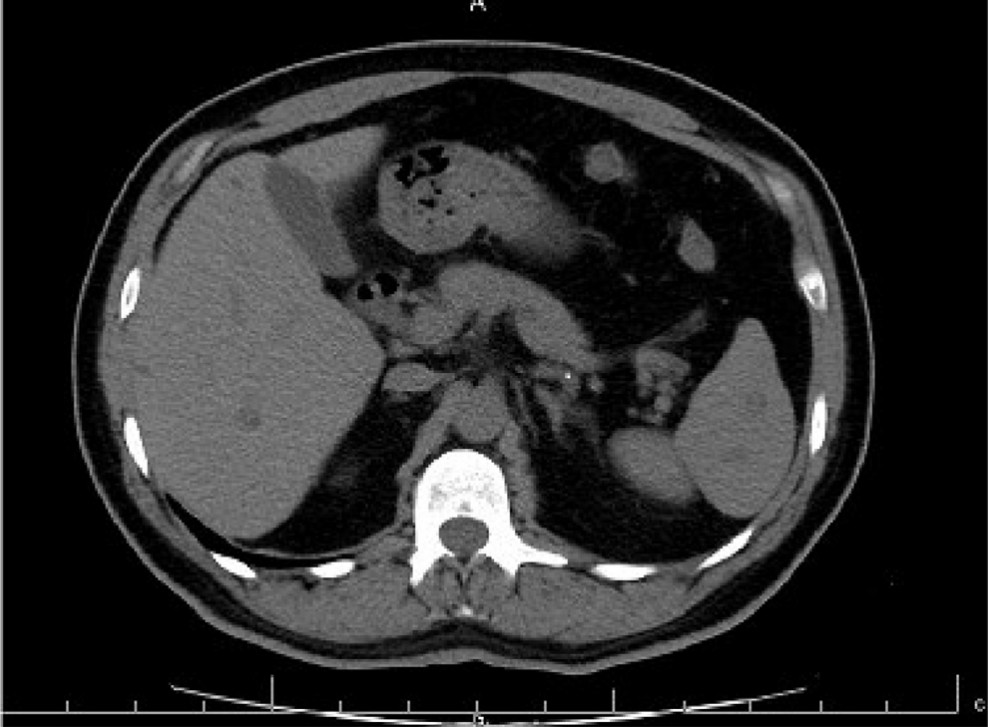
A round heterogeneous lesion which is hypodense in comparison with liver parenchyma, with density more than water measured up to 7 mm in the liver. It may contain internal echo or thin septations on ultrasound.

**Fig. 2:**
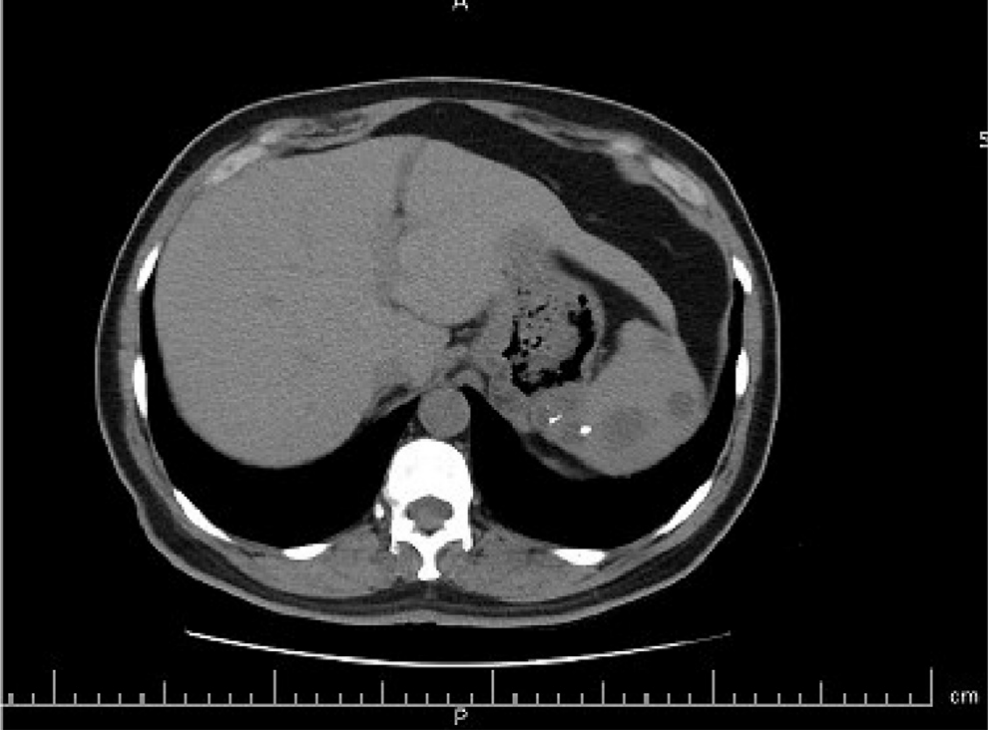
Multiple hypodense lesions in spleen with some internal and wall calcifications measured up to 27 mm.

**Fig. 3:**
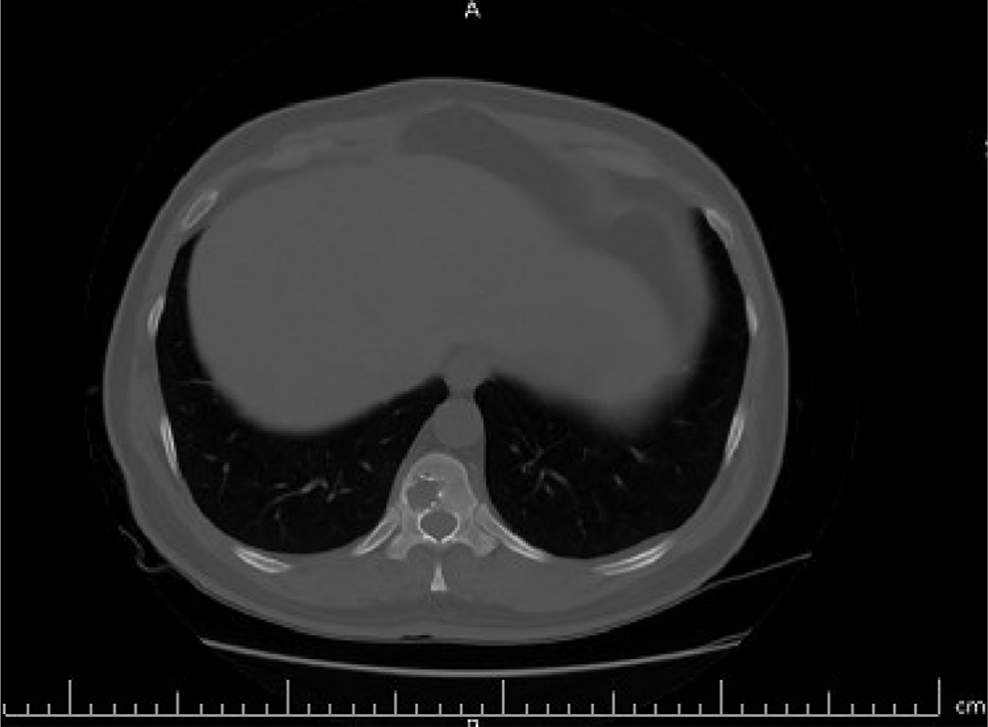
A lytic lesion in the 12th thoracic vertebral body, without cortical destruction.

**Fig. 4:**
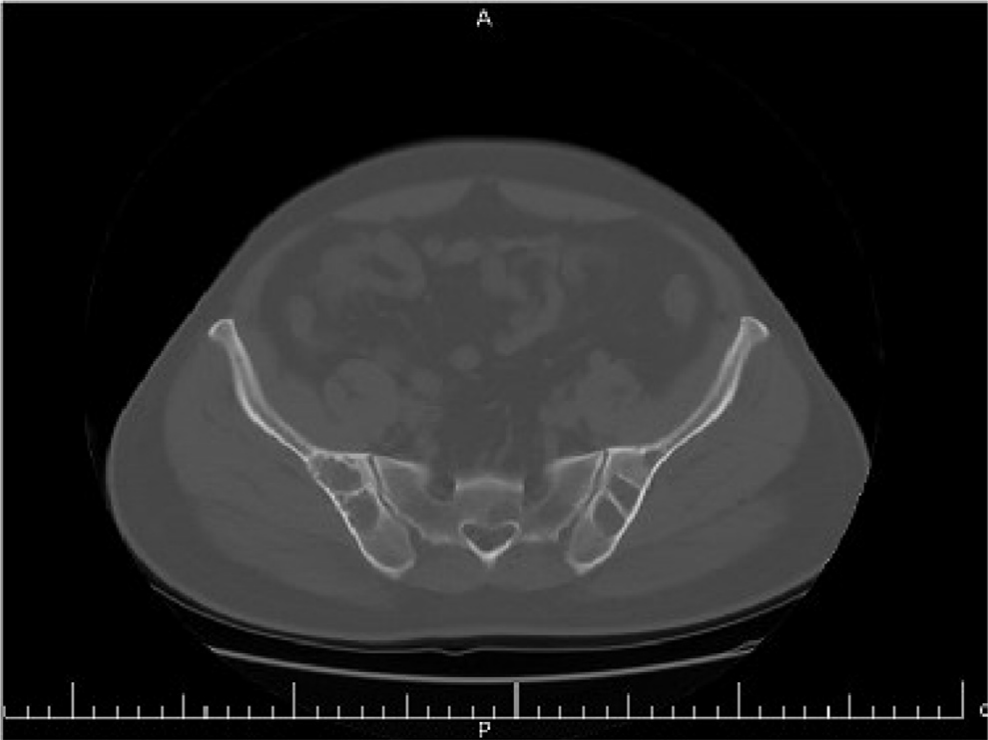
Multilocular lytic lesions without soft tissue component and extraosseous extension, in bilateral iliac bones in the vicinity of sacroiliac joints.

The case study is devoted to investigating left flank pain and hematuria in a 42-year-old male who presented to emergency department (ED) of our institution (Hasheminejad Hospital) in Tehran, Iran. After clinical examinations done by an emergency physician, the first differential diagnosis for him was renal colic; hence he underwent an abdominopelvic CT scan without contrast. We assessed the performed CT scan and noticed mild left hydroureteronephrosis and a 3mm stone in the submucosal part of the left ureterovesical junction that could explain the patient's clinical symptoms. But we also noticed some hypodense lesions with water density measured up to 7mm in the liver and also spleen with some internal and wall calcifications measured up to 27 mm (Figs. 1 and 2). Also, there was a lytic lesion in the 12th thoracic vertebral body and some multilocular lytic lesions without soft tissue component and extraosseous extension [8], in bilateral iliac bones in the vicinity of sacroiliac joints (Figs. 3 and 4). In limited sections of the chest CT scan, there was a cystic lesion containing calcified areas in the left para cardiac region. We called the patient to ask about symptoms and medical history, he denied any other symptoms except left flank pain and hematuria. He mentioned that he had had cervical surgery because of his cervical mass when he was a child, without a definite diagnosis and no documents.

We put all these findings together along with his previous cervical mass, and our first differential diagnosis was generalized lymphangiomatosis. Also, Lymphoma could be a differential diagnosis, but it was ruled out because of the presence of calcification within the lesions. We need more evaluations such as a complete chest CT scan, pelvic magnetic resonance imaging (MRI), and biopsy.

**Fig. 5:**
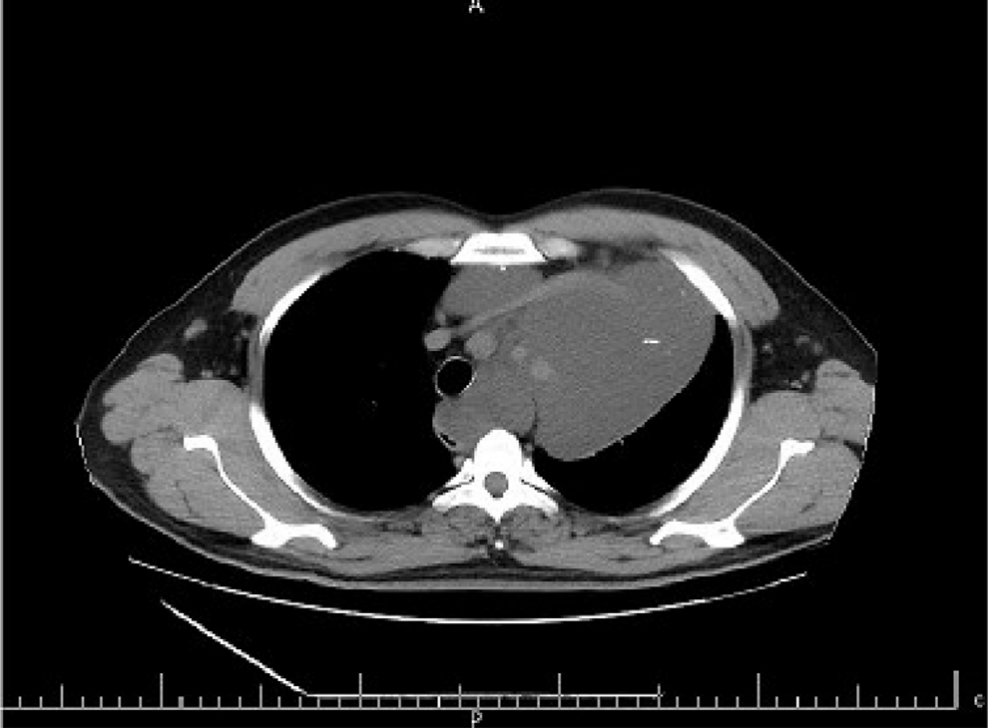
A large well-defined cystic lesion containing calcification in the anterior superior mediastinum. This lesion caused thoracic aorta encasement. Extension of the lesion to the left paracardiac region is noted.

**Fig. 6:**
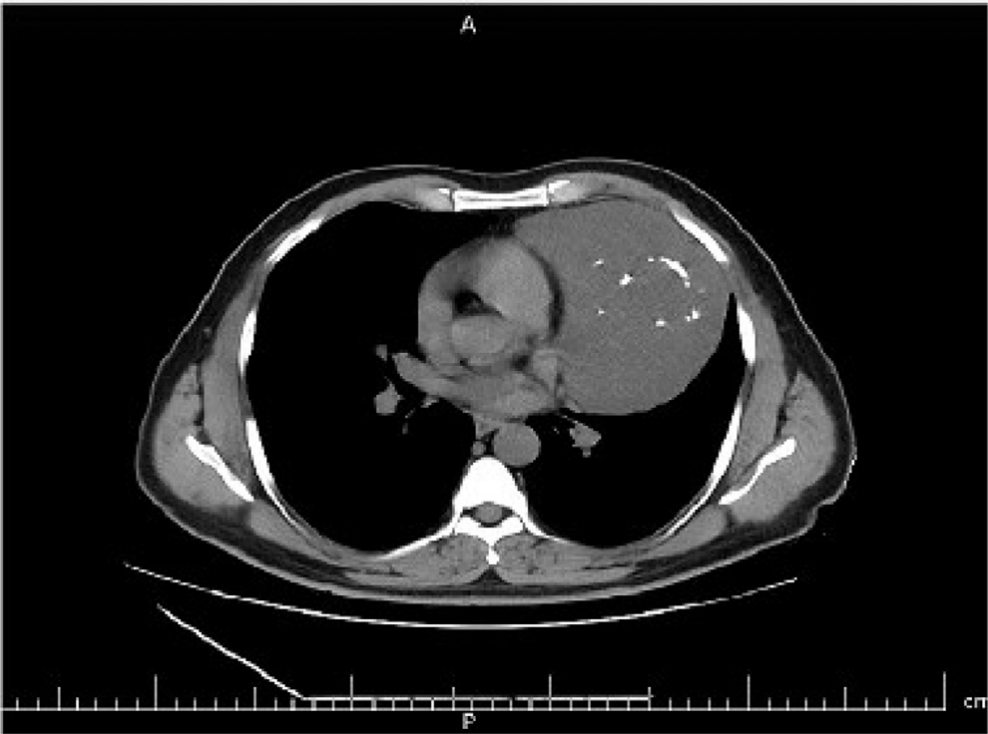
A large well-defined cystic lesion containing calcification in the anterior, superior, and inferior mediastinum. This lesion caused thoracic aorta encasement. Extension of the lesion to the left paracardiac region is noted.

**Fig. 7:**
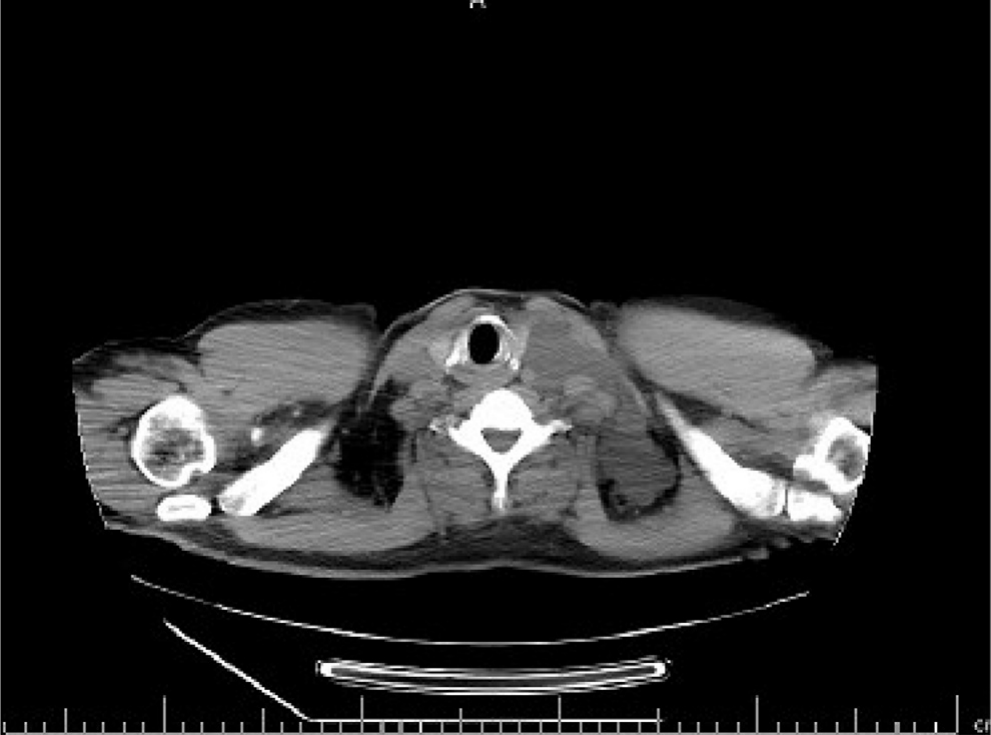
Extension of the mediastinal mass to the left posterior triangle of the left neck is also noted.

In the chest CT scan, there was a large well-defined cystic lesion containing calcification in the anterior superior mediastinum. This lesion caused thoracic aorta encasement. Extension of the lesion to the left para cardiac region and posterior triangle of the left neck was also noted (Fig. 5, Fig. 6, Fig. 7). The above findings are in favor of massive lymphangioma. Some lytic lesions in 5th and 12th thoracic vertebral bodies and the posterior arch of the 4th rib were also seen, which could be bony lymphangioma, considering the presence of lymphangioma in the other organs.

**Fig. 8:**
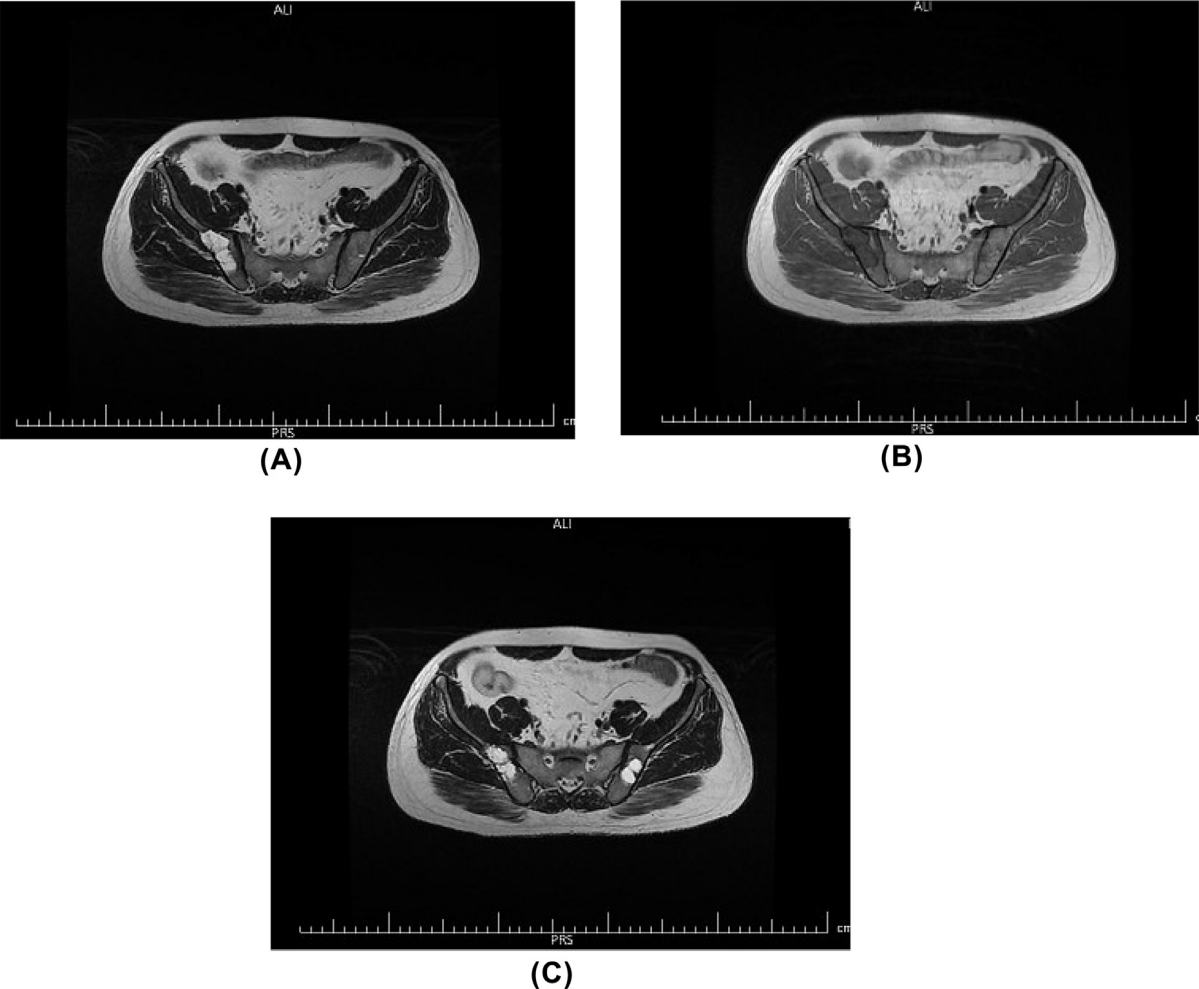
(A–C) In the pelvic MRI, there were 2 well-defined multiseptated cystic lesions in bilateral iliac bones which show high signal intensity on T2 and low signal intensity on T1 sequence. No cortical disruption or soft tissue component was visible. Regarding patient history, findings are compatible with macro cystic lymphangioma.

In the pelvic MRI, there were 2 well-defined multiseptated cystic lesions in bilateral iliac bones which show high signal intensity on T2 sequence. No cortical disruption or soft tissue component was visible. Regarding patient history, findings are compatible with macro cystic lymphangioma (Figs. 8(A)–(C)).

The patient underwent CT guided biopsy of the mediastinal lesion, and the pathological evaluation revealed fibro adipose tissue with some empty vascular channels lined by flat endothelium and several mature-looking small lymphocytes in the granular proteinaceous background. The impression was a benign vascular lesion, in favor of lymphangiomas, and negative for malignant cells.

## Discussion

Lymphangiomas are benign lesions arising from proliferated lymphatic vessels most commonly found in the head and neck. They can originate from any tissue except ocular and neural tissue and are described to be congenital lymphatic malformations causing lymphatic obstruction and the development of lymphangiectasia [7,8]. Up to 65% of patients are children and infants and almost 90% of cases are diagnosed within the first 2 years of life, and it is very uncommon in adults. It occurs with an equal gender prevalence and there is no reported correlation with familial factors [9]. Lymphangiomatosis is described as a unique and rare pathological condition in which multiple lymphangiomas are present, mostly in the liver, spleen, mediastinum, or lungs [3]. They contain endothelial-lined spaces surrounded by connective tissue stroma of variable thickness containing lymphoid tissue, smooth muscles and round cells. Focal areas of adipose tissue, lymphocytes, and phleboliths may also detected [3,9,6,10]. The symptoms often are abdominal pain, gastrointestinal bleeding, and protein-losing enteropathy or a palpable abdominal mass. However many patients are asymptomatic and are diagnosed incidentally on abdominal imaging, typically, as a multiloculated cystic lesion with septa. Also in ultrasound, it may be present as an anechoic lesion due to the fluid contents, which represents blood, pus, or chyle [3,10].

CT scan and MRI give us more information about the size, extension, and relationship to adjacent structures. A CT scan may reveal non-enhancing well-demarcated multiseptated cystic lesions with low attenuation internal density [11]. However, MRI has demonstrated several advantages compared to other radiologic imaging modalities, especially in the detection of bone and soft tissue lesions [8]. This pattern is similar in soft tissues and bony structures, but sharper demarcations with a sclerotic rim are more prominent in bone lesions [12]. Pelvic bones, femur, ribs, vertebra, and skull are the most affected bones in generalized lymphangioma [13,14]. Osseous involvement occurs mostly as lytic lesions, but sclerotic lesions have also been reported very rarely [15]. Typically, the bone involvement pattern is well-defined, round lytic lesions with sclerotic margins without periosteal reaction and soft tissue component [10]. Recently, MRI is widely used for the better diagnosis of generalized lymphangiomatosis. It can characterize the lesion morphology better and can clarify the lesion origin. Previous studies show that lytic lesions of lymphangiomatosis typically appear hypointense on T1-weighted MRI and hyperintense on T2-weighted images, while the rare sclerotic type is seen as hypointense on both T1- and T2-weighted images [15,16]. In our patient, the clinical history was concerning for renal colic, so an abdominopelvic CT scan was done to evaluate the genitourinary system, but incidentally, multiple hypodense lesions were detected in the liver and spleen. Also, a large cystic lesion in the mediastinum with extension to the para cardiac region and left neck and lytic lesions in vertebral bodies, ribs, and iliac bones were detected. Putting these findings together with the history of a cervical mass in childhood, suggested generalized lymphangiomatosis, confirmed by pathologic assessment.

Recently, many treatment options were introduced for this condition, such as medical therapies like sirolimus, propranolol, and sildenafil. Also, other therapies like MEK inhibition, irradiation, surgery, and sclerotherapy were introduced [17].

Generalized lymphangiomatosis in adults has been reported very rarely in the literature. Marom et al. reported generalized lymphangiomatosis, In a 30 year-old-man who had been observed for 24 years for slowly growing chest wall masses [15]. The difference between our patient with previously reported cases is that our patient has been asymptomatic for many years and the disease was diagnosed incidentally in an abdominopelvic CT scan done to evaluate renal colic.

## Conclusions

Generalized lymphangiomatosis is a rarely reported disease in children and young adults. Delayed diagnosis in older patients or misdiagnosis is common due to its rarity and nonspecific clinical presentation. In this paper, we aimed to emphasize on early diagnosis of the disease with imaging modalities, especially for asymptomatic patients. Radiologists should be aware that they should know the manifestations of the disease in different modalities such as ultrasound, CT scan, and MRI to diagnose the disease sooner and help the clinician to start the therapy if needed (Table 1).

**Table 1:** Summarizing the findings from various diagnostic tools for diagnosis of lymphangiomatosis.

Imaging modality	Manifestations
**Ultrasound**	Hypodense lesions in solid organs such as liver and spleen with water density that may contain internal echo. They also may contain septations or wall calcifications.
**CT Scan**	CT scan may reveal nonenhancing well-demarcated multiseptated cystic lesions with low attenuation internal density in organs such as liver, spleen, mediastinum and neck. This pattern is similar in soft tissues and bony structures, but sharper demarcations with a sclerotic rim are more prominent in bone lesions
**MRI**	Lytic lesions of lymphangiomatosis typically appear hypointense on T1-weighted MRI and hyperintense on T2-weighted images, while the rare sclerotic type is seen as hypointense on both T1- and T2-weighted images.

## Author contributions

AD chose the case, reported the CT scan and MRI and supervised manuscript preparation. ZHT reviewed the published literature and wrote the case report manuscript. SHS interviewed the patient and collected the patient's past medical records.MM edited the final manuscript. All authors read and approved the final manuscript.

## Patient consent

I declare that the patient is fully informed about the research and give me permission in full consciousness to use photographs, clinical and laboratory data of patient. Written informed consent is taken from patient. Anonymous use of patient's data was approved by the ethics committee of Hasheminejad Kidney Centre.
